# Physicians’ Mutual Benefit Association of Texas; Mrs. Harrington’s Benefit, Receipt, and List of “Paid”

**Published:** 1888-10

**Authors:** 


### SOCIETY NOTES.

### PHYSICIANS' MUTUAL BENEFIT ASSOCIATION OF TEXAS.

Assessment No. 7, for the benefit of the heirs of Dr. Y. D. Harrington, was issued July 21. Although there were only one hundred and twenty-six members in good standing—twenty-one of the 147 who were on the roll at the time having failed to pay Mrs. Glover's benefit, the notices were sent to all, including the twenty-one delinquents. Of this number, the following only paid, one hundred and twenty-five in all. Deducting the cost of the postal cards and printing, stamps or cards for receipts and second notices, premium on exchange, etc.,—$5 in all, there was $120 net cash, which was promptly forwarded to Mrs. Harrington. Below is published her receipt.

TERRELL, TEXAS, OCTOBER 12, 1888.

Received of Dr. W. A. Morris, President, and F. E. Daniel, Secretary and Treasurer Physicians' Mutual Benefit Association of Texas, one hundred and twenty dollars, collected under assessment No. 7, for benefit of Dr. Harrington's certificate No. 47.

[Signed.] MRS. MARY HARRINGTON.

LIST OF MEMBERS WHO PAID NO. 7:

F. E. Daniel, J. W. Daniel, Burts, Wolff, Sholars, Cupples, Herff, Spring, Swearingen, Day, J. W. Dupre, W. J. Dupree, Duchien, Towsey, Coffey, T. M. Mathews, W. J. Mathews, Sterrett, Paine, Brown, Styles & Styles, Myers, Powell, M. A. Taylor, W. A. Taylor, Hardy, Weller, McFarland, Inabnet, Paulus, Webb, Gray, Ball, Reid, Strain, Tidwell, Hill, Morris, Bennett, Ross, Francis, Thorpe, Throckmorton, Hors, Scales, Perkins, Keating, Osborn, Mayfield, Ford, Murph, Q. C. Smith, Bat Smith, Tinsley, Litten, McClintock, Fox, W. G. Jameson, J. Y. T. Jameson, Martin, Gregg, Richmond, Wilson, A. D. Evans, A. H. Evans, Frazer, Field, Wadgyman, Fennell, Shuford, Burke, Bowers, Patton & Patton, Denman, Tally, Lancaster,

Watlington, May, Getzwiller, Foster, McGehee, Wakefield, Stinson, Fry, Kaiser, Bell, McCreary, Southworth, Witherspoon, Jones, Bass, Musick, Bundy, Davies, Hamilton, Cain, Barbee, Nicholson, Starnes, Beck, Burrows, Burroughs, Vawter, Harrison & Harrison, Robinson, Maddox, Bruce, Grace & Grace, Phillips & Phillips, Felder, Reeves, Harmer, Seymour, Beckman, Hewson, Oliver, Sams, L. B. Johnson, W. H. Wilkes, R. A. Taylor—125.

